# Applying a Consumer Behavior Lens to Salt Reduction Initiatives

**DOI:** 10.3390/nu9080901

**Published:** 2017-08-18

**Authors:** Áine Regan, Monique Potvin Kent, Monique M. Raats, Áine McConnon, Patrick Wall, Lise Dubois

**Affiliations:** 1 School of Public Health, Physiotherapy and Sports Science, University College Dublin, Dublin 4, Ireland; patrick.wall@ucd.ie; 2 School of Epidemiology and Public Health, Faculty of Medicine, 600 Peter Morand Crescent, University of Ottawa, Ottawa, ON K1G 5Z3, Canada; Monique.PotvinKent@uottawa.ca (M.P.K.); ldubois@uottawa.ca (L.D.); 3 Food, Consumer Behaviour and Health Research Centre, University of Surrey, Guildford GU2 7XH, UK; m.raats@surrey.ac.uk; 4 Avila Research, Cabinteely, Dublin 18, Ireland; aine.mcconnon@gmail.com

**Keywords:** consumer behavior, multi-actor, reformulation, salt reduction

## Abstract

Reformulation of food products to reduce salt content has been a central strategy for achieving population level salt reduction. In this paper, we reflect on current reformulation strategies and consider how consumer behavior determines the ultimate success of these strategies. We consider the merits of adopting a ‘health by stealth’, silent approach to reformulation compared to implementing a communications strategy which draws on labeling initiatives in tandem with reformulation efforts. We end this paper by calling for a multi-actor approach which utilizes co-design, participatory tools to facilitate the involvement of all stakeholders, including, and especially, consumers, in making decisions around how best to achieve population-level salt reduction.

## 1. Introduction

Across the globe, governments have adopted healthy eating strategies to reduce the incidence of diet-related non-communicable diseases [1]. The reduction of population-level salt intake has been singled out as a ‘best buy’ feasible and cost-effective public health initiative by the World Health Organisation (WHO). There is consistent evidence showing that reductions in population salt intake leads to reductions in blood pressure which subsequently lowers the risk for cardiovascular disease [2,3,4]. Debate over this relationship has been amplified through recent media reporting of conflicting science; however, the evidence in favor of salt reduction remains convincing (see Webster et al. 2017 for a comprehensive review on this topic). In 2013, WHO member states agreed on a global target of a 30% reduction in mean population intake of salt by 2025 [5]. Achieving this target will require a multi-actor approach combining many different elements and strategies.

As of 2014, 75 countries were identified as having national salt reduction strategies, representing a 50% increase from 2010 [3]. Of these strategies, 80% incorporated some element of food industry engagement, and reformulation of food products to reduce salt in particular was a common strategy [6]. Food reformulation has been identified as a key pillar for achieving population level salt reduction [7], particularly in industrialized countries where processed and restaurant foods are the largest contributor of total individual dietary sodium intake [6]. In contrast, reformulation efforts are not prioritized in countries such as China and Japan, where salt added during cooking/at the table is a larger contributor to sodium in the diet than processed foods [6]. For those countries engaging the food industry in salt reduction efforts, a voluntary and collaborative reformulation approach has been favored. This generally involves the publication of reduction benchmarks to guide food manufacturers in reducing levels of unhealthy target nutrients voluntarily. In some countries, reformulation efforts are accompanied by a coordinated monitoring and evaluation program, with the goal of holding the food industry accountable for achieving reformulation targets [8]. That said, an increasing number of countries are opting for legislative means to limit the salt levels in foods, for example, through mandating maximum levels of sodium content in specific foods, taxing high-sodium products and mandatory food labeling schemes [3]. Modeling studies suggest that legislative measures are more effective than voluntary reformulation programs in reducing consumers' salt intake [9]. At the same time, there is evidence to suggest that the voluntary approach is also making progress. Across several countries with voluntary programs, significant reductions have been made in the salt content of commonly consumed foods [6]. For example, in the UK, a comparison between 2006 and 2011 showed a 7% reduction in the overall mean sodium content of foods measured [10]. Across government and industry, there has been relatively widespread stakeholder acceptance of reformulation as a feasible and cost-effective strategy for reducing population level salt intake.

With processed foods identified as contributing between 75–80% of total sodium intake in many industrialized countries, reformulation as a strategy to reduce population-level salt intake has resulted in part from a belief that consumers are unable to adequately monitor or change their own salt intake [11]. Indeed, recent research highlights that, while consumers are aware of high levels of salt content in processed food, they may still underestimate the extent of those salt levels [12]. By adopting a strategy of making changes to the environment as opposed to trying to change the behavior of the individual, reformulation attempts to overcome the well-acknowledged difficulty of achieving positive behavior change amongst consumers who are aware of the negative impact of salt on health, but who often fail to take action [13]. Reformulation strategies predominantly focus on changing the food environment rather than consumer behavior; in this paper, we argue that understanding and accounting for consumer behavior remains central to the success of reformulation strategies and salt reduction initiatives more broadly. We also argue the merits of having consumers more centrally involved in designing and implementing salt reduction initiatives to ensure that factors which shape consumer behavior are identified and addressed from the outset.

## 2. Accounting for Consumer Behavior in Salt Reduction Initiatives

Even though a salt reduction initiative such as reformulation is not primarily focused on changing consumer behavior, consumer behavior still has an important role in determining the ultimate success of that initiative. Whether a consumer decides to accept or reject a reformulated product will ultimately determine the success of reformulation strategies. Significant attention within the area of reformulation has been given to the technical food science and technology developments required to reformulate foods and to the regulations which need to be considered [14]. Reformulation efforts are heavily underpinned by an evidence base of food and sensory science research to ensure the technical quality and safety of the reformulated products [15]. However, without consumer acceptance, these efforts are futile. In the following section, we consider how reformulation strategies can be impacted by consumer behavior and what we can learn from this to ensure the future success of salt reduction initiatives.

A key challenge for reformulation efforts is determining the best strategy to introduce reformulated products onto the market; anticipating how consumers will react to reformulated foods is critical to this issue. Some argue that foods should be reformulated gradually without making the consumer aware of such activity, adopting the argument that population taste will adjust in line with this gradual reformulation effort [15]. To be successful in shifting population tastes and maintaining consumer acceptance, a principle of progressive, incremental change is usually followed. There is consistent evidence to suggest that preference for dietary salt can be adapted following reduction in the salt content over a period of time [11,16]. This reduction can be achieved through modest, sequential reductions over a relatively short period of time, even as little as six weeks [17]. Often, industry stakeholders prefer to make these changes without informing consumers, a strategy commonly referred to as ‘health by stealth’ [13]. Reformulation of a product is not actively promoted in the belief that this will prevent consumers from rejecting products based on the perceived attributes of the product (e.g., perceiving that a reformulated product is of reduced taste or lower quality) [1]. Food manufacturers are concerned that if consumers are made aware that a product has been reformulated to include less salt, they may assume that it will have inferior sensory properties because they psychologically associate ‘low salt’ with poor taste or quality.

The stealth approach is not without its challenges. Eventually, the food industry will reach a point at which ‘stealth’ reformulation will no longer be a viable option; on the one hand, technical challenges will be faced whereby further salt reduction could compromise the safety of the product and on the other hand, consumers will reach a threshold where they will begin to notice a sensory change in the product [13,18]. The latter in particular is a concern for the food industry. This was evident in relation to the highly-publicized 2011 Campbell’s Select Harvest Soup range, where the company announced that they were going to renege on reformulation commitments and add salt back into their soup formulations in an effort to boost declining sales [15]. While further research in the area of sensory science and food technology can help to address these concerns to some extent, user-led research could also offer significant value to reformulation efforts [19]. For example, co-design approaches with consumers to decide what category of foods are acceptable to target for further reformulation could be of particular interest; for example, food with a currently healthy image may be more acceptable for reformulation than unhealthy foods [20]. Co-design approaches would help to elucidate the likely future success of further reformulation efforts as well as the preference amongst consumers for replacement ingredients and consumer acceptance of food technology processes used to replace sodium in foods. This is a particularly salient point as behavioral research has demonstrated consumer aversion towards perceived ‘unnatural’ technology processes and a demand for ‘clean labels’—foods with minimal and natural ingredients lists [21].

To adjust and ‘train’ consumer palates for a low-salt diet, widespread buy-in from all members of the food industry is required such that widespread salt reduction efforts exist within similar-product categories and across different-product categories. For example, if only some food manufacturers reduce the amount of salt in, for example, bread, and other higher-salt content breads are still available on the market, then it will be difficult for consumers to adjust their sensory acceptance of low-salt bread [13]. However, even where salt reductions are made simultaneously across all products in a certain food category, this will still not be sufficient to achieve change. A whole-of-industry approach is required to ensure that consumers’ overall diets are substantially lower in salt; reducing salt content in one category of foods will have little impact unless salt reductions occur across a sufficiently large number of different product categories [15,18]. If such a scenario is ever to be realized, consumer demand for a low-salt food value chain is necessary—product supply needs to align with consumer demand. For example, there is evidence to suggest that reducing salt in commercial food could simply lead to consumers adopting compensatory behavior and adding it back in through discretionary salt use at preparation and consumption [22]. In contrast, if efforts are made to encourage the consumer to seek a low-salt ‘way of life’, this would lead to more successful reformulation efforts: previous research has found that consumers who are motivated to reduce their own dietary salt intake are then more accepting of actual reductions in the salt content of food products [15]. Reformulation efforts need to be coupled with strategies to motivate and facilitate individual consumer behavior change.

There has been much attention focused on nudging consumers towards healthier food choices via food labeling [13]. In their audit of food labeling across five European countries, Hieke et al. [23] identified that 26% of the products sampled displayed a nutrition or health claim or a symbol identifying products as healthier options for consumers. Such claims and symbols play a key role in influencing consumers’ perceptions and expectations not only about the product, but also about the manufacturer. Labels allow manufacturers to promote the positive nutritional characteristics of food products and to demonstrate to the consumer a commitment and willingness to be transparent in their reformulation efforts [24]. It is common for reformulation strategies to be carried out in tandem with front-of-pack (FOP) labeling, mandatory nutrition labeling, and/or social marketing campaigns to encourage consumer demand for healthier food products [25,26]. Although sensory attributes drive much of the food industry’s approach to food reformulation, and undoubtedly are primary indicators of acceptability of a reformulated food product, food selection and consumption is also influenced by non-sensory factors such as an individual’s health and dietary needs, social relationships and their general attitudes towards health and wellness [27]. Many food manufacturers have tapped into the increasing market demand for more healthful food products; product reformulation is viewed as giving them a competitive advantage as it allows them to market their products as healthier [25,26]. It has been reported that the turning point in the salt reduction campaign in the UK was when salt actually became a competitive marketing issue between companies [28].

Nutrition claims are the labels which bear most relevance for food reformulation efforts, as they allow food manufacturers to market their food products as healthier versions of the original product. Nutrition claims can take the form of a content claim that describes the level of the nutrient contained in the food or a comparative claim, where the label compares the nutrient levels and/or energy value of two or more similar foods [20]. It is these latter claims which make it explicit to the consumer that reformulation has taken place (e.g., ‘30% less sodium’ or ‘reduced salt’). The ability to promote their reformulation efforts via comparative claims may act as an incentive for food manufacturers to engage in reformulation—but it is not always straightforward. For example, Buttriss [21] relates the point that under European regulations, manufacturers must achieve at least a 30% reduction in salt to enable them to make comparative nutrition claims on product labeling, which is a substantial change and would significantly alter the product, potentially losing consumers—even those who are motivated to seek out low-salt products. Comparative claims can also give the impression to the consumer that the salt level of the product was much too high to begin with, calling into question the overall healthiness of the food product [29]. Indeed, the impact of comparative claims on consumers’ perceptions and behaviors has been found to have undesirable effects: research shows that when exposed to products with ‘reduced salt’ labeling, consumers revealed immediate negative taste perceptions and engaged in compensatory salt use, adding additional salt to the meal [30]. Comparative claims, although a seemingly attractive option for marketing industry reformulation efforts, also may have unintended negative behavioral effects.

There is also a moral argument as to whether it is appropriate for inherently unhealthy foods to be labeled and marketed as ‘low salt’ or ‘reduced salt’. Evidence suggests that such labels could lead to a halo effect whereby the consumer assumes the food to be, on the whole, a healthier choice than it actually is, in essence, allowing consumers to interpret such labels as a license to (over)indulge [31,32]. Rather than focusing on individual nutrient content, labeling which communicates at the level of the healthiness of the overall food through simplified nutrition labels and symbols on the front of the pack may be more desirable. Labeling systems which interpret the nutrient profile of the whole food have been implemented in different countries including the Dutch Choices logo, ‘Pick the Tick’ and the ‘Health Star Rating’ in Australia and New Zealand, and the American Heart Association’s heart-check mark [33,34]. To be allowed to carry such health logos/labels, the food must have a nutrient composition which complies with criteria for maximum levels of different nutrients including saturated fat, sugar and sodium. In order to meet these criteria for processed foods and obtain a better overall nutrition profile, reformulation may be required, thus, these symbols can act as a motivator for industry to reformulate [35,36]. Warning labels can also act as an incentive for industry to reformulate; for example, New York City adopted a mandatory sodium warning label policy on restaurant menus whereby a warning symbol had to accompany a meal which had more than 2300 mg of sodium [37].

In choosing different labeling options to accompany reformulation strategies, assumptions are being made about how people view food and make decisions related to health. In particular, an assumption is being made about whether consumers consider healthfulness of food at the individual nutrient level (e.g., salt) or whether they make decisions based on the overall perceived healthiness of the product, bringing many different attributes to bear on their decision. It is vital to reflect on how consumers process labeling information and their preferred formats for receiving nutrition information. Evidence suggests that inconsistencies in labeling across a variety of different schemes can leave consumers confused about what particular figures or symbols represent, or the recommended daily intake for specific nutrients [38]. Having to compare food products which vary in their levels of unhealthy nutrients can also challenge consumers’ information processing capabilities [39]. When it comes to making purchasing decisions, consumers tend to engage in heuristic information processing which involves ‘skimming’ the available information in a situation and using cues (prior experience, familiar brand) for quick judgments [40]. Interpreting food labeling is often viewed as difficult and time-consuming and it is often the case that consumers will only engage with food labeling information when there is a particular motivation to do so [41,42]. Labeling can act as a motivator and facilitator for some consumers to purchase healthier food products. It can also incentivize reformulation amongst food manufacturers. However, we cannot depend on consumers to pay attention to labeling in every situation; thus, it alone is not sufficient to supplement reformulation efforts. Both initiatives need to be coupled with wider policy incentives and supports, and importantly, there is a need to ensure that the views and voices of consumers are considered at each step of policy development.

## 3. Future Directions and Conclusions

Significant action has been taken to reduce population-level salt intake; however, the recommended global intake level of less than five grams of salt per day has not yet been achieved [29] and meeting the global target of a 30% reduction in mean population intake of salt by 2025 will require more action [5]. Reformulation has been identified as a key pillar for achieving these targets [7]. However, its potential impact should be contextualized. For example, a number of Asian countries have the highest global sodium intakes but the primary dietary source is not processed foods, rather salt added during cooking [43,44]; coupled with the size of the population in this region, to achieve a reduction in global salt intake, initiatives which reach beyond reformulation efforts will be necessary. At the same time, in industrialized countries, behavioral research shows that we cannot focus only on making changes to the food environment—we also need to account for how the consumer will adapt and react to those changes. In order to reach set targets, a multi-faceted approach towards salt reduction is recommended [6]. As laid out in this paper, this approach should be executed with careful consideration of how the consumer will respond and react to salt reduction initiatives. In the current paper, we have referred to relevant behavioral research to support the presented arguments; however, we have not carried out a systematic review of the literature. Therefore, our paper does not reflect the full breadth of work in this field and some relevant literature may not have been included. Future research would be well placed to undertake a systematic review of the available literature to further support, clarify or contest the arguments laid out in the current paper.

Reaching global salt reduction targets will require a multi-actor approach comprising of government, public health agencies, the food manufacturing industry, the restaurant and catering industry, scientists, health care professionals, and consumers working together [13]. Future research is needed to better understand the different viewpoints which drive the reformulation agenda and more broadly, the process through which salt reduction policies and strategies are developed [45,46]. In so doing, this will help to build a better understanding of the extent to which current strategies are being developed to meet the needs of consumers and whether more can be done to ensure that the voice of the consumer is being accounted for during the policy-making process. Greater engagement of consumers in policy development can help to build mutual trust and reflexivity. There has been much discussion on the introduction of healthy eating policies by governments. In the UK, the Responsibility Deal has generated debate over the appropriate role of government in encouraging food environment changes. Consumers may not be as receptive to public health initiatives as we might anticipate—concerns over restricted food choices and ‘nanny-state’ sentiments have been evident in the past [47,48]. A recent study with Irish consumers found that a large majority of the respondents were, at least in principle, supportive of reformulation strategies to reduce salt intake—however, as policies became more restrictive, support declined [45]. Including consumers in the design of initiatives could be a mechanism for ensuring that salt reduction strategies have consumer buy-in, whilst also ensuring fairness and transparency—key principles for good governance [49]. There is significant merit in considering how we can better involve the consumer at an earlier stage of policy development with regard to salt reduction strategies. Co-design and participatory approaches and tools can help to facilitate the involvement of all stakeholders, including, and especially, consumers, in the design and implementation of salt reduction strategies.

Through reformulation, significant reductions to the salt content of commonly-consumed foods have already taken place, although further reductions are required. However, reformulation alone will not suffice; ultimately, future success will require a concerted effort by all stakeholders along the value chain. There is a need to strengthen accountability structures so that key stakeholders shoulder an appropriate level of responsibility for taking action to reduce salt intake. The food industry needs to continue in their efforts to lower the salt content of food products and to adopt responsible and transparent labeling; consumers need to be encouraged to reduce their discretionary use of salt and to purchase products that are low in salt; policy-makers need to implement policies which target the range of determinants contributing to a high salt diet including individual and environmental factors; public health agencies and health care professionals need to continue their efforts in driving and supporting consumer behavior change. Actions by stakeholders across the value chain will need to be implemented at a regional and national level in a manner that is culturally appropriate and that seeks and anticipates the views and responses of the end user: the consumer.
